# Sharing feelings online: studying emotional well-being via automated text analysis of Facebook posts

**DOI:** 10.3389/fpsyg.2015.01045

**Published:** 2015-07-23

**Authors:** Michele Settanni, Davide Marengo

**Affiliations:** ^1^Department of Psychology, University of TurinTurin, Italy; ^2^Department of Social Sciences and Humanities, University of Aosta ValleyAosta, Italy

**Keywords:** emotional well-being, psychological measurement, psychological assessment, social networking sites, cyberpsychology, psychoinformatics

## Abstract

Digital traces of activity on social network sites represent a vast source of ecological data with potential connections with individual behavioral and psychological characteristics. The present study investigates the relationship between user-generated textual content shared on Facebook and emotional well-being. Self-report measures of depression, anxiety, and stress were collected from 201 adult Facebook users from North Italy. Emotion-related textual indicators, including emoticon use, were extracted form users’ Facebook posts via automated text analysis. Correlation analyses revealed that individuals with higher levels of depression, anxiety expressed negative emotions on Facebook more frequently. In addition, use of emoticons expressing positive emotions correlated negatively with stress level. When comparing age groups, younger users reported higher frequency of both emotion-related words and emoticon use in their posts. Also, the relationship between online emotional expression and self-report emotional well-being was generally stronger in the younger group. Overall, findings support the feasibility and validity of studying individual emotional well-being by means of examination of Facebook profiles. Implications for online screening purposes and future research directions are discussed.

## Introduction

Social network sites (SNS), such as Facebook and Twitter, have experienced a dramatic increase in popularity over the last few years (Morrison, 2014).

Facebook, in particular, has reached a leading position among SNS, with a number of worldwide active users amounting to over 1.3 billion as of July 2014 (Statistic Brain, 2014). There are indications that accessing Facebook has become a daily activity for many SNS users: 48% of them log on the site at least once a day, 98% of those aged 34 or less visiting their Facebook profile as soon as they wake up (Statistic Brain, 2014).

One specific feature of Facebook and others SNS is the possibility for users to share self-generated content such as texts, pictures, audio, and video clips with their online social networks. Given the nature of communication processes on SNS, user-generated content is open to feedback and further dissemination by other users, resulting in a massive network of inter-connected data.

For social scientists, digital traces of user activity on SNS represent a vast and relatively new source of ecological data with potential connections with individual behavioral and psychological characteristics (Barbier and Liu, 2011; Tov et al., 2013).

In recent years, an increasing number of studies have addressed the relationship between user behaviors on Facebook and psychological constructs, with personality differences being one of the most active areas of research (Mehdizadeh, 2010; Ryan and Xenos, 2011; Kosinski et al., 2013; Winter et al., 2014). Significant links between Facebook behaviors and indicators of emotional well-being have also been reported (Kramer, 2010; Moreno et al., 2011; Fernandez et al., 2012; Chen and Lee, 2013; Park et al., 2013; Rosen et al., 2013; Wright et al., 2013; Bevan et al., 2014; Schwartz et al., 2014), indicating the potential use of Facebook-related data to identify people with specific risk profiles (e.g., individuals at risk for depression). A serious limitation of many of these studies is the use of indirect measures of Facebook behaviors obtained solely via self-report questionnaires (Chen and Lee, 2013; Rosen et al., 2013; Wright et al., 2013; Bevan et al., 2014), as opposed to the examination of actual online behaviors. Some other studies relied on the assessment of actual Facebook behaviors by means of inspection of Facebook profiles, generally focusing on user-generated textual data (Kramer, 2010; Moreno et al., 2011; Fernandez et al., 2012; Park et al., 2013; Lin et al., 2014; Schwartz et al., 2014). This approach is based on the assumption that posts and comments shared on SNS can be seen as entries of a traditional diary or journal, in the sense that they may reflect tastes, behaviors, attitudes, and beliefs of the users (Sinn and Syn, 2013).

For some of these researches, the inspection of profiles was manually performed by trained personnel (Moreno et al., 2011; Fernandez et al., 2012). Although capable to provide detailed and controlled data, this approach is extremely expensive and inefficient, thus unsuitable for the examination of large populations. Recent studies have explored the feasibility of studying psychological constructs via the automated extraction and analysis of textual data posted by users on SNS profiles (Golder and Macy, 2011; Park et al., 2012; Wang et al., 2012; De Choudhury et al., 2013; He et al., 2014; Lin et al., 2014; Schwartz et al., 2014). Studies employing this approach on traditional non-online sources (e.g., diaries, transcripts of clinical consultations, speeches, and interviews) have shown significant links between emotion-related word use and measures of depression (Mehl, 2006; Rodriguez et al., 2010; Tov et al., 2013) and other symptoms of psychological distress, such as anxiety (Bekker et al., 2003; Hofmann et al., 2012; Ahmad and Farrell, 2014), and stress (Pirzadeh and Pfaff, 2012; Tov et al., 2013). Results from studies exploring the relationship between linguistic indicators extracted from Facebook and emotional well-being, however, have been mixed and inconclusive (Kramer, 2010; Wang et al., 2012; Schwartz et al., 2014). Indeed, these studies reported only weak-to-moderate relationship between psychological and linguistic indicators: this is possibly due to the use of non-validated instruments and the lack of consideration of relevant moderating variables, such as users’ characteristics influencing motives of SNS use (Panek et al., 2013; Davenport et al., 2014; Hollenbaugh and Ferris, 2014) and behaviors. For example, the amount of self-disclosure exhibited by users on SNS has been shown to vary with age: Young SNS users are generally more prone to share personal information (Christofides et al., 2011; Denti et al., 2012), use self-references, and show different patterns of emotional expression compared to older people (Pfeil et al., 2009). Similar results emerged from research on other media, such as online blogs (Herring et al., 2004; Argamon et al., 2007). Studies also showed the presence of age-specific motives for SNS, with younger adults using Facebook as a means of communicating with others and maintain oﬄine relationships (Hayes et al., 2015). Facebook usage by older adults is more influenced by narcissistic motives, such self-promotion and image management (Davenport et al., 2014). The presence of these differences suggests the need for social researchers to include age as a possible intervening variable in studies based on SNS data. To our knowledge, until now, no studies investigated the influence of age differences on the relationship between user-generated content posted on SNS and psychological constructs.

### Aims of the Study

Our aim is to investigate the relationship between emotion-related linguistic indicators derived from text corpora collected from Facebook profiles (namely, users’ status updates and comments), and self-report measures of depression, anxiety, and stress.

As an additional aim, we examined age differences in both the textual content shared on Facebook and the relationship between online emotional expression and self-report emotional well-being. Our hypothesis is that, given the existence of differences in both motives and Facebook use, this relationship may differ in significance and direction across age groups.

## Materials and Methods

### Participants

The sample for this study consisted of adult volunteers, mostly recruited online using a snowball sampling procedure. We recruited a starting seed of 20 Italian university students, aged 18 or more, from different universities in the North of Italy. A specific research page was created including a Facebook-sharable link to an online questionnaire. Participants were required to answer the questionnaire and add the Facebook page of the research PI as a Facebook friend. This step was needed to collect information from participant Facebook profiles. We collected informed consent from all participants prior to the administration of the online questionnaire. We even invited participants to share the link to the research page with their Facebook friends, provided they were at least 18 years old. Out of 366 individuals who answered the online questionnaire, 165 persons were not included in the final sample because they either did not add the research page as Facebook friend or their privacy setting on Facebook did not allow the collection of status updates. This yielded a final sample of 201 participants (66% females) with a mean age of 28.4 (SD = 7.3). In order to investigate possible biases due to significant differences between actual participants and the 165 respondents who were not included in the final sample, we conducted *t*-tests on age and self-report measures: no differences emerged between the two groups.

### Procedure and Measures

#### Facebook Data Extraction

The Microsoft Excel 2013 Power Query module was employed to collect the participants’ Facebook textual data. For each participants, we collected the status updates and associated comments posted during the last 12 months and integrated them in single text corpora. For the purpose of this study, only comments posted by the participants were included in the text corpora, while we discarded comments posted by their friend. Overall, 28,595 posts (status updates and comments) were collected, with an average of 143.69 posts (SD = 81.87) per participant, with an average word count of 2485.54 words (SD = 1849.80). On average, the proportion of words published in status updates was 68.9 %, while words published in comments accounted for the remaining 31.1%.

#### Linguistic Inquiry and Word Count (LIWC) Emotion Coding of Facebook Textual Data

Automated text analysis was performed on the participants’ Facebook data with the Linguistic Inquiry and Word Count (LIWC) software (Pennebaker et al., 2007), which is one of the most used software by social scientists for this kind of analyses. LIWC includes a semantic dictionary including 64 categories measuring emotional, cognitive, and structural components contained in text-corpora on a word-by-word basis. LIWC’s validity and reliability on a variety of indicators is supported by several studies in different contexts and languages (Alpers et al., 2005; Bantum and Owen, 2009; Tausczik and Pennebaker, 2009). The Italian version of the 2001 LIWC dictionary was used (Alparone et al., 2004). For the purpose of this study, we performed analyses on emotion-related categories (positive and negative emotions) and their subcategories – i.e., optimism, anger, anxiety, and sadness. For each emotion-related LIWC category examples of words are reported in **Table 1**. For an extensive description of the LIWC categories, see the LIWC software documentation (Pennebaker et al., 2007).

**Table 1:** Examples of words of the Linguistic Inquiry and Word Count (LIWC) categories.

LIWC category	Example words
Positive emotions	Happy, pretty, good
Optimism	Ease, trust, hope
Negative emotions	Hate, worthless, enemy
Anxiety	Nervous, afraid, tense
Anger	Hate, kill, pissed
Sadness	Grief, cry, sad

In order to include emoticons in the text analysis performed with LIWC, we added two new customized categories to the LIWC dictionary referring, respectively, to positive and negative emoticon sentiments according to the classification proposed by Vashisht and Thakur (2014) (see **Table 2**).

**Table 2:** Examples of emoticons and associated sentiment.

Emoticons	Sentiment
:-)	Positive
=)	Positive
:D	Positive
:p	Positive
:’(	Negative
>: (	Negative
: (	Negative
:/	Negative

#### Self-Report Measures

##### Demographic variables

Participant demographics were collected and included gender and age. In order to investigate age differences, participants were grouped according to the cut-off age of 25, resulting in two groups consisting of, respectively, 90 young adults aged ≤25 (*M* = 22.7, Median = 23, SD = 2.0, Range = 18–25) and 111 older adults aged >25 (*M* = 33.2, Median = 32, SD = 6.7, Range = 26–60). The proposed age threshold is based on social and developmental psychology literature which have documented differences between this age group and older adults with respect to their general psychosocial development (Arnett, 2007) and specifically to their patterns of use of technology and social media (e.g., Coyne et al., 2013; Satici and Uysal, 2015; Vaterlaus et al., 2015). Further segmentation of the sample by age was not performed due to the positive skewness of the age distribution. Preliminary analyses revealed no differences between the two age groups concerning number of posts and average word count [# of posts: *t*(199) = 0.57, *p* = 0.57; word count: *t*(199) = 1.23, *p* = 0.22]. In order to rule out differences in the recency of posts we examined the distribution of posts and comments published in the four trimesters prior to the questionnaire administration. A chi-square goodness-of-fit test, comparing the observed distribution of posts to a uniform distribution yielded a non-significant result [χ^2^(3) = 3.47, *p* = 0.32]. Further, no significant differences in the proportion of posts published in the four trimesters emerged when comparing the two age groups [χ^2^(3) = 1.12, *p* = 0.77].

##### Emotional well-being

An adapted version of the DASS-21 questionnaire was administered to collect information about emotional well-being. The DASS-21 is an internationally validated self-report instrument including three scales measuring depression, anxiety, and stress (Henry and Crawford, 2005); for the purpose of the present study, the Italian version was used (Severino and Haynes, 2010). Each scale includes seven items. Items consist of statements referring to the past months, and each item is scored on a 4-point scale, ranging from 0 (*Did not apply to me at all*) to 3 (*Applied to me very much*, or *most of the time*). Scale scores are computed by summing the scores of the associated items. In our sample, Cronbach’s alphas were 0.83 for anxiety, 0.88 for depression, 0.84 for stress, and 0.92 for the total score. Example items are: “I felt I was close to panic,” “I was worried about situations in which I might panic and make a fool of myself” (Anxiety); “I couldn’t seem to experience any positive feeling at all,” “I found it difficult to work up the initiative to do things” (Depression); “I found it hard to wind down,” “I was intolerant of anything that kept me from getting on with what I was doing” (Stress).

### Data Analysis Strategies

Descriptive statistics were preliminary computed on all the study measures. We performed a set of *t*-tests for independent samples in order to investigate mean differences in the study variables between age groups. We computed Pearson correlation coefficients to examine the relationship between the LIWC categories and the DASS-21 scales for the total sample and by age groups. Significant correlations were further analyzed using Fisher’s *r*–*z* transformation (alpha level = 0.05) to test the significance of the correlation differences among the age groups.

## Results

### Descriptive Statistics and Mean Differences Across Age Groups on the Emotional Measures

**Table 3** presents the descriptive statistics relative to the study measures both for the total sample and by age groups. Results of the *t*-tests across age groups are also reported. Significant differences emerged on emoticon use, on both LIWC positive and negative emotions categories and on the sadness subcategory, with participants aged 25 years or less reporting significantly higher scores than the older ones. No significant differences emerged as regards the DASS-21 scales.

**Table 3:** Descriptive statistics and mean differences across age groups on the LIWC and self-report emotional measures.

	Age > 25 (*n* = 111)		Age 18–25 (*n* = 90)			Total	
	*M*	SD	*M*	SD	*t*	*M*	SD
**LIWC codings**
Positive emotions	1.16	0.49	1.32	0.60	-2.08*	1.23	0.54
Optimism	0.55	0.36	0.56	0.26	-0.23	0.55	0.32
Negative emotions	1.53	0.83	1.79	0.86	-2.17*	1.65	0.85
Anxiety	0.26	0.74	0.24	0.20	0.25	0.25	0.56
Anger	0.43	0.31	0.53	0.45	-1.86	0.47	0.38
Sadness	0.56	0.34	0.71	0.35	-3.07**	0.63	0.35
Positive emoticons	0.92	1.20	1.56	1.38	-3.51**	1.21	1.35
Negative emoticons	0.07	0.14	0.17	0.25	-3.60**	0.11	0.20
**DASS-21**
Anxiety	2.21	2.99	2.74	2.99	-1.25	2.45	2.99
Depression	4.63	4.02	4.49	4.18	0.24	4.57	4.08
Stress	6.48	3.71	6.52	3.72	-0.08	6.50	3.71
Total score	13.32	9.51	13.76	9.30	-0.33	13.52	9.39

*p < 0.05, **p < 0.01.

### Correlations

**Table 4** shows the correlations between study variables for the overall sample. We found significant positive correlations between the negative emotions LIWC categories and all the DASS-21 scales. In particular, sadness was positively correlated with both the overall and subscales scores, while anger was only significantly correlated with the overall score and anxiety subscale. Even the emoticons presented significant correlations: positive emoticons was negatively related to the total score and the stress subscale, while negative emoticons positively correlated with the anxiety subscale. As a whole, correlations were modest, ranging from -0.15 to 0.34.

**Table 4:** Correlations between LIWC emotion codings and DASS-21 measures (*N* = 201).

	DASS-21			
LIWC codings	Total score	Anxiety	Depression	Stress
Positive emotions	0.02	0.09	-0.01	-0.00
Optimism	-0.13	-0.09	-0.11	-0.13
Negative emotions	0.22**	0.26**	0.14*	0.19**
Anxiety	-0.01	0.01	-0.02	-0.00
Anger	0.14*	0.20**	0.10	0.09
Sadness	0.32**	0.34**	0.22**	0.29**
Positive emoticons	-0.14*	-0.07	-0.14	-0.15*
Negative emoticons	0.07	0.14*	0.03	0.02

*p < 0.05, **p < 0.01.

As shown in **Table 5**, different correlation patterns emerged across age groups. More specifically, when compared to the older group, correlations were generally stronger among participants aged 25 or less, although differences in correlations among age groups were not always significant. In the younger group, both negative emotions and sadness indicators positively correlated with all the DASS-21 scales, while anger showed a moderate positive correlation with the total score and the anxiety subscale. Moreover, positive emoticon use negatively correlated with the DASS-21 total score and the stress subscale. Concerning the older group, the sadness subcategory positively correlated with all the DASS-21 scales, except for depression. No other significant correlations emerged. Significant differences in the strength of the correlations with the DASS-21 scales (Total score, depression, and stress) emerged between age groups and concerned, respectively, the LIWC negative emotions, anger, and anxiety indicators; correlations that showed significant differences among age groups (*p* < 0.05) are reported in bold in **Table 5**. Age differences approaching statistical significance (0.05 < *p* < 0.10) were also found between self-report anxiety and both LIWC negative emotions and anger.

**Table 5:** Correlations between LIWC emoticon coding and DASS-21 measures by age group.

	DASS-21			
LIWC codings	Total score	Anxiety	Depression	Stress
**Age > 25 (*N = 111*)**
Positive emotions	-0.01	0.13	-0.07	-0.05
Optimism	-0.17	-0.10	-0.16	-0.18
Negative emotions	0.06	0.14	-0.02	0.06
Anxiety	-0.05	-0.03	-0.04	-0.06
Anger	-0.04	0.07	-0.14	-0.02
Sadness	0.26**	0.29**	0.17	0.25**
Positive emoticons	-0.08	-0.02	-0.09	-0.09
Negative emoticons	0.08	0.15	0.06	0.01
**Age 18–25 (*N = 90*)**
Positive emotions	0.05	0.03	0.05	0.05
Optimism	-0.06	-0.08	-0.03	-0.06
Negative emotions	0.40**	0.37**	0.33**	0.34**
Anxiety	0.19	0.19	0.07	0.25*
Anger	0.31**	0.30**	0.30**	0.19
Sadness	0.40**	0.39**	0.30**	0.35**
Positive emoticons	-0.23*	-0.17	-0.18	-0.22*
Negative emoticons	0.06	0.11	0.03	0.02

orrelations showing significant (*p* < 0.05) difference in strength across age groups are in bold.

*p < 0.05, **p < 0.01.

## Discussion and Conclusion

The main aim of the present study was to investigate the relationship between emotion-related linguistic indicators extracted from Facebook posts and self-report measures of emotional well-being of adult Facebook users.

Findings show the presence of significant correlations between LIWC emotion-related categories and users’ emotional well-being. Overall, the expression of negative emotions positively correlated with anxiety, depression, and stress symptoms. More specifically, the highest correlation was found between sadness expression and all the facets of psychological distress. At the same time, anger expression positively correlated with anxiety. A significant negative correlation was found even among the use of positive emoticons and stress symptoms. Instead, negative emoticon use positively correlated with anxiety symptoms. These findings are congruent with what reported by studies examining text corpora from other sources, such as online blogs, diaries (Rodriguez et al., 2010; Tov et al., 2013), and spoken natural language transcriptions (Mehl, 2006).

Findings from our study also suggest that online emotional expression and its relationship with self-report emotional well-being may vary across age groups. Overall, young adults showed a higher frequency of use of both positive and negative emotion-related words and emoticons than older adults did. Moreover, the relationship between online emotional expression and self-report well-being was generally stronger for the young adult group than for older adults. In particular, the most noteworthy difference regards the correlations between the expression of negative emotions and self-report emotional well-being. The correlations were moderate in the young adult sample, while they were not significant in the older group, even if not all the correlation differences among age groups reached statistical significance. Only sadness expression significantly correlated with self-report emotional well-being in both groups. Another significant finding concerns the use of anger-related words. There were no differences as regards anger expression between the two age groups. Still, anger word use correlated with emotional well-being only in the younger group. With respect to emoticon use, young adults used them more frequently. Further, a relevant association between use of positive emoticons and psychological distress was only found in the young adults group, even if the difference in correlation strength among age groups was negligible. Overall, this finding, even if it is not generalizable to the entire population, seems to indicate that, among SNS users, emoticon use is more strongly linked with actual emotional well-being than indicators based on actual words expressing positive emotions.

The lack of significant relationships between use of positive emotional words and emotional well-being is consistent with previous studies (Liu et al., in press), and may be related to the use of impression management strategies. On the other side, positive emoticon use, being a more direct, less controlled, kind of emotional expression, may be more strongly linked with self-report emotional well-being than the use of positive emotional words. Further studies are needed to deepen our understanding of the differences among these diverse ways to express positive emotions in social media.

As a whole, our findings highlight the presence of age-related differences in the level of actual emotional self-disclosure on Facebook. This result is congruent with previous findings (Pfeil et al., 2009; Gibson et al., 2010) and can be linked to differences in motives and patterns of Facebook use, such as the desire of older people to avoid display of negative emotions to not worry loved ones (Gibson et al., 2010) or as an impression management tactic. As regards the difference in the strength of the relationship with emotional well-being, a possible interpretation of this result is that young adults may be more prone to freely express themselves on SNS (Manago et al., 2012), thus producing content that more accurately mirrors their personal characteristics. At the same time, older adult Facebook users may be more concerned with privacy issues (Christofides et al., 2011), thus posting content less closely related to their actual inner feelings and emotions. However, more research is needed to clarify the causes of these phenomena.

Findings from our study support the feasibility and validity of studying individual emotional well-being by means of examination of Facebook profiles. In particular, it is important to note that: (1) Textual indicators extracted from Facebook profiles may be used to study emotional well-being in an ecologically valid, naturalistic context; (2) Given the popularity of Facebook and the relative ease and low cost of data collection procedure, our approach could be extended to study large samples in a cost-effective way; (3) The possibility to collect data at different time-points allows the implementation of longitudinal researches aimed at studying change in psychological constructs over time (e.g., by tracking individual trajectories); (4) A similar approach could be used to develop online screening instruments for the identification of users at risk for psychological distress; (5) Our results highlight that caution should be exercised when applying this kind of approach to older populations.

The main limitation of our study regards the sample, which is non-probabilistic and limited in number. This is due to the exploratory nature of this study: more data should be collected to consolidate its findings. The small sample size even affected the possibility to apply more advanced analytical methods, such as those based on Machine Learning (e.g., Wald et al., 2012; Kosinski et al., 2013), which would require a significantly larger dataset. Another limitation linked to the sample was the use of self-selection sampling. Included participants could be more comfortable than average in sharing personal information, potentially influencing the strength of the relationship between language use and well-being. This is a common limitation of studies of this type, which is difficult to overcome. Further studies are needed to explore the differences among individuals who differ with respect to their propensity to let researchers access information they publish on SNS. Lastly, the present study employed only measures of self-report negative well-being. The use of indicators of positive well-being – e.g., happiness, life satisfaction – would have provided further insights into the relationship between self-expression on SNS and the actual emotional state of SNS users.

## Future Steps

Next steps in our research program will be threefold. First, we will deepen the age differences issue, by means of collecting and analyzing more data from both adolescents and older populations, even looking for the determinants of the relationships among word use and personal characteristics. Secondly, we will investigate possible differences in the strength of the relationships with well-being of emotional expressions among different types of posts, namely status updates and comments. Lastly, we will focus on the relationships between topics expressed on Facebook and psychological constructs and behaviors. This will require both a larger dataset and the adoption of specialized text mining techniques, such as Latent Semantic Analysis (Landauer et al., 1998) and Latent Dirichlet Allocation (Blei et al., 2003).

## Conflict of Interest Statement

The authors declare that the research was conducted in the absence of any commercial or financial relationships that could be construed as a potential conflict of interest.
